# A Novel Prognostic Scoring System of Intrahepatic Cholangiocarcinoma With Machine Learning Basing on Real-World Data

**DOI:** 10.3389/fonc.2020.576901

**Published:** 2021-01-20

**Authors:** Zhizhen Li, Lei Yuan, Chen Zhang, Jiaxing Sun, Zeyuan Wang, Yu Wang, Xin Hao, Fei Gao, Xiaoqing Jiang

**Affiliations:** ^1^ Department of Biliary Tract Surgery I, Eastern Hepatobiliary Surgery Hospital, Shanghai, China; ^2^ Winchester School of Art, University of Southampton, Southampton, United Kingdom; ^3^ Department of Medicine, Beijing Medicinovo Technology Co., Ltd., Beijing, China; ^4^ School of Computer Science, University of Sydney, Sydney, NSW, Australia; ^5^ Department of Medicine, Dalian Medicinovo Technology Co., Ltd., Dalian, China

**Keywords:** intrahepatic cholangiocarcinoma, prognosis, staging system, machine learning, overall survival

## Abstract

**Background and Objectives:**

Currently, the prognostic performance of the staging systems proposed by the 8th edition of the American Joint Committee on Cancer (AJCC 8th) and the Liver Cancer Study Group of Japan (LCSGJ) in resectable intrahepatic cholangiocarcinoma (ICC) remains controversial. The aim of this study was to use machine learning techniques to modify existing ICC staging strategies based on clinical data and to demonstrate the accuracy and discrimination capacity in prognostic prediction.

**Patients and Methods:**

This is a retrospective study based on 1,390 patients who underwent surgical resection for ICC at Eastern Hepatobiliary Surgery Hospital from 2007 to 2015. External validation was performed for patients from 2015 to 2017. The ensemble of three machine learning algorithms was used to select the most important prognostic factors and stepwise Cox regression was employed to derive a modified scoring system. The discriminative ability and predictive accuracy were assessed using the Concordance Index (C-index) and Brier Score (BS). The results were externally validated through a cohort of 42 patients operated on from the same institution.

**Results:**

Six independent prognosis factors were selected and incorporated in the modified scoring system, including carcinoembryonic antigen, carbohydrate antigen 19-9, alpha-fetoprotein, prealbumin, T and N of ICC staging category in 8th edition of AJCC. The proposed scoring system showed a more favorable discriminatory ability and model performance than the AJCC 8th and LCSGJ staging systems, with a higher C-index of 0.693 (95% CI, 0.663–0.723) in the internal validation cohort and 0.671 (95% CI, 0.602–0.740) in the external validation cohort, which was then confirmed with lower BS (0.103 in internal validation cohort and 0.169 in external validation cohort). Meanwhile, machine learning techniques for variable selection together with stepwise Cox regression for survival analysis shows a better prognostic accuracy than using stepwise Cox regression method only.

**Conclusions:**

This study put forward a modified ICC scoring system based on prognosis factors selection incorporated with machine learning, for individualized prognosis evaluation in patients with ICC.

## Introduction

Intrahepatic cholangiocarcinoma (ICC) is a malignant neoplasm originating from the epithelial cells of bile ducts located above the secondary bile duct branch (1). It is the second most common primary malignancy of liver and its incidence has been increasing in recent years (2–4). Surgical resection is the main potentially curative for ICC, the 5-year overall survival (OS) rates after hepatectomy and lymphadenectomy is 15 to 35% (5–9). Appropriate staging for ICC patients can be used to describe the severity and range of involvement of malignant tumors, thus prompting clinicians to understand the prognosis of the disease.

Now the eighth edition of American Joint Committee on Cancer (AJCC 8th) staging system and the Liver Cancer Study Group of Japan (LCSGJ) staging system are widely used in clinical practice (10–13). Although studies have demonstrated that the modified AJCC staging system improves stratifying ability, it remains controversial (14, 15). The LCSGJ staging system focuses on the hepatocellular carcinoma (HCC) which has distinct differences in biological behaviors and postoperative outcomes (16). Some new stratification strategies begin to incorporate readily available clinical parameters, such as carbohydrate antigen 19-9 (CA19-9), alkaline phosphatase (ALP) and alpha-fetoprotein (AFP) (17–19). To more effectively utilize these clinical parameters, not just on surgical-pathological factors, we combined the robust machine learning methods to analyze the high-dimension data in clinical practice.

Meanwhile, the selection of variables which involved in the outcome imputation was significant for staging performance. In similar studies, multivariate analysis using Cox regression to identify the independent prognostic factors for survival was a common method, such as the ICC prognostic staging systems performed by Zhou et al. (19), the modified staging system for mass-forming ICC (16), the Fudan score (17), and in nomogram predicting strategies (18). In present study, we attempted to improve the conventional survival analysis by combining with machine learning algorithms for variable selection, since in the real-world studies, variables are not always independent to each other and they are closely related in the non-linear way. The normal used multivariate analysis methods or linear models cannot capture the complex relationships of variables, which are machine learning methods skilled in, especially we used decision tree-based ensemble methods, i.e., eXtreme Gradient Boosting (XGBoost), random forest (RF), and gradient boosted decision tree (GBDT). The three methods are able to divide and re-aggregate the variables to achieve the minimum prediction error when growing sub-trees. Through this way, the non-linear relationship between variables can be well captured. In addition, they are all with the ability of learning from data with missing values directly, that can better adapt to the data situation in the real world. To confirm their effectiveness, we performed the three variable selection methods for comparison and our proposed method outperforms others by a significant margin. Moreover, our study also incorporated the prognostic factors for TNM staging as an improvement of traditional strategy.

The objective of the current study is to integrate pathological factors and clinical parameters to construct a useful and personalized scoring system with machine learning methods, which can accurately predict the survival outcomes of ICC patients under surgical resection.

## Materials and Methods

### Patients Cohort

The cohort comprised 1,390 pathologically confirmed ICC patients who underwent hepatectomy between January 2007 and October 2015 at the Eastern Hepatobiliary Surgery Hospital (EHBH) in Shanghai, China, which is a high-volume medical center. The data collection was cut-off on November, 2018. Patients diagnosed with Perihilar (Klatskin) tumors and mixed with hepatocellular carcinoma tumors were excluded. All deaths were confirmed to have occurred after ICC recurrence to avoid the interference of competing mortality. The data collection and tumor staging processes were supervised and examined by two pathologists. The patients in external validation cohort (n=42, January 2016 to June 2017) were screened with the same criteria of the internal cohort. The data collection was cut-off on June, 2020. Variable characteristic statistics of the training cohort and external validation cohort were summarized in **Supplemental Table** and **Supplementary Data of Entire Cohort**. The protocol of this study has been approved by the Ethics Committee of the EHBH, and the informed consent has been exempted in the Ethical approval documents.

We collected data of 27 clinical independent variables including provided basic clinical information (age, gender, jaundice, history of stone, history of tumor, and smoking), laboratory results [blood type, hepatitis B virus (HBV), CA19-9, γ-glutamyltranspeptidase (γ-GT), albumin (Alb), alanine aminotransferase (ALT), ALP, prealbumin (PA), aspartate aminotransferase (AST), carcinoembryonic antigen (CEA), AFP, direct bilirubin (DBIL), and total bilirubin (TBIL)], and perioperative data (T/N/M or TNM stage in AJCC 8th, T or TNM stage in LCSGJ, resection type, and tumor size). All laboratory examinations were performed within 1 week before resection or intervention. To be applicable to machine learning, all relevant variables were cleansed and converted into numerical codes.

### Study Design

The aim of this research was to construct a more accurate and simple ICC scoring system for predicting the prognosis after resection based on the clinical factors and stages. Overall Survival for 3 years after resection was the end point in our study. We enriched many types of variables in the initial cohort, and variable selection was implemented *via* three machine learning methods, i.e., XGBoost, RF, and GBDT. The algorithms calculated the contribution of each independent variable to the target variable and obtained the importance score (IS). We combined the intersection variables with the highest IS for further analysis.

**Figure 1:**
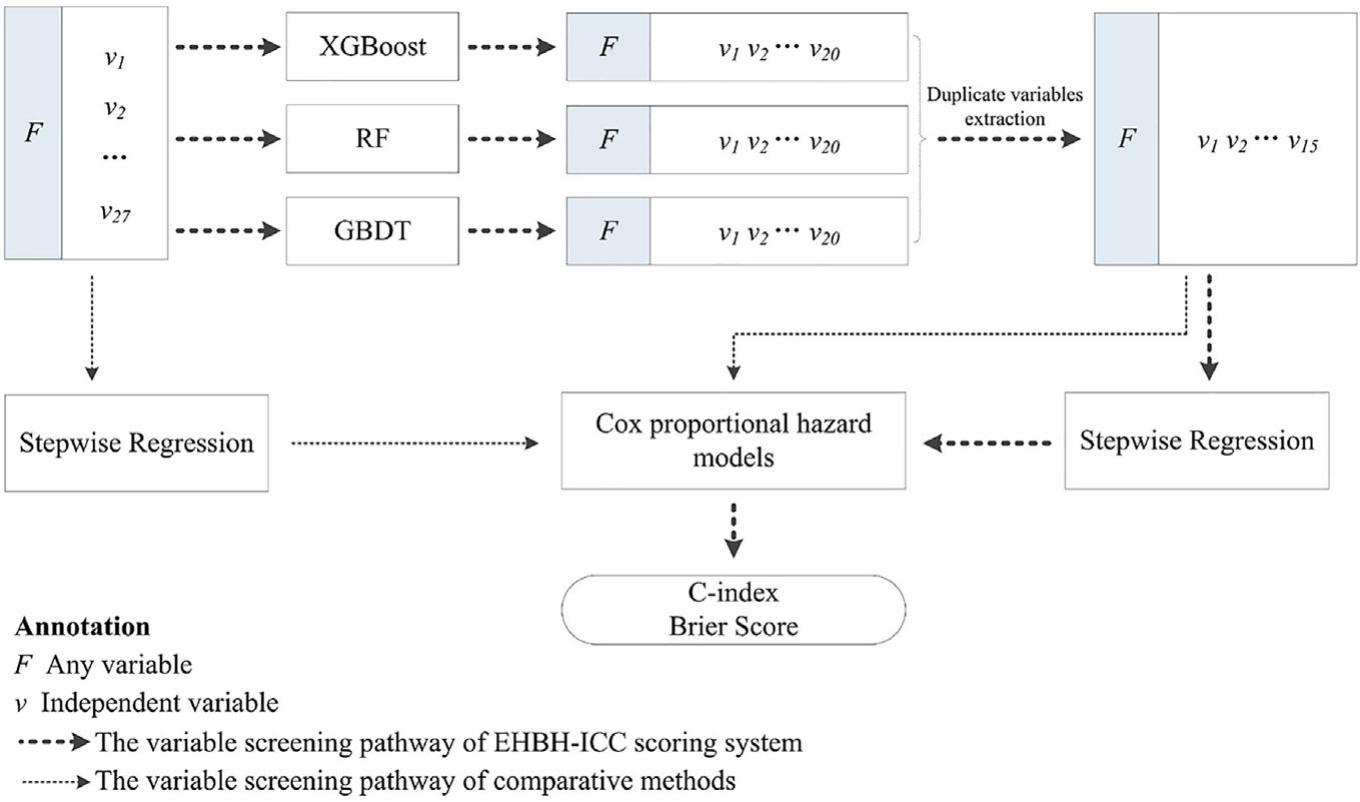
The workflow of this study.

Cox proportional hazard models with backward stepwise regression were used to evaluate the impacts of intersection variables on survival, and the prognostic scoring equation was obtained. Overall, the predictive accuracy and discrimination ability between models were compared. In addition, for validating the advantages of the research methods, we compared survival predictions with/without machine learning screening. Since the data collection and research were implemented in the Eastern Hepatobiliary Surgery Hospital (Shanghai, China), this scoring strategy we proposed is simply called EHBH-ICC in the later section. The overall study process is illustrated in **Figure 1**.

### Tumor, Node, Metastasis Stage

The 8th edition of AJCC and the LCSGJ staging manual in patients who underwent operations were adopted as baseline models for performance comparison (1, 20).

### Machine Learning

In the process of machine learning modeling, we chose the XGBoost, RF, and GBDT for the variable selection, which are capable of dealing with missing values under certain assumptions and do not require data imputation. Since our data was derived from real-world settings with a small number of missing values, machine learning methods with incomplete data learning ability are necessary. We performed these three algorithms using Scikit-learn: a machine learning framework (https://www.scikit-learn.org/stable/) in Python 3.6.8. In order to achieve their best performance, the AutoML (https://github.com/ClimbsRocks/auto_ml) method was adopted to automatically select their hyperparameters.

### Statistical and Survival Analysis

Data statistics were characterized as quantity (%) or median (interquartile range, IQR). Mann-Whitney U test and chi-square were used on continuous variables and categorical variables respectively, and *p*<0.05 was considered statistically significant. Relevant prognostic predictors were evaluated by the Cox proportional hazard model using backward stepwise regression (Wald-test, *p*<0.05 represents a significant difference). We ensured comparability of the training and internal validation cohorts, a random distribution was applied in a ratio of 8:2. To estimate the influence of prognostic factors, the hazard ratio (HR) was calculated. Kaplan-Meier analysis was used in survival analysis and log-rank test was adopted to compare significant differences. The Concordance Index (C-index) and Brier Score (BS) were utilized to evaluate the discrimination ability and predictive performance of the staging methods. The higher C-index indicates, the better discrimination ability of the model. BS was an important measure of model calibration, i.e., the mean squared difference between the predicted probability and the actual outcome. The lower BS value indicates the higher prediction accuracy of the model. Statistical analysis and modeling were performed using Python (version 3.6.8) and R Studio (version 1.1.463).

## Results

### Clinicopathologic Characteristics of Patients

A total of 1,390 patients underwent surgical resection for ICC during the study period. Twenty-seven types of variables included in the primary entire cohort were sorted out and input into the models, patients’ demographic information, medical history, tumor information, and examination information were contained in modeling and reported in **Table 1**. The median survival time was 15.5 months (IQR 7.7 to 27.7 months). Of all ICC patients in this study, there were 560 of them (40.3%) having a survival of less than 1 year, 576 patients (41.4%) died between 1 and 3 years after surgery, while 254 (18.2%) died after 3 years. There were 939 females (67.6%) and 451 males (32.4%) enrolled in the study, with a male-to-female ratio of 1:2.1. Among study population, 316 patients (22.7%) had HBV infection. TNM staging and T classification of AJCC 8th and LCSGJ were evaluated. The T classification (AJCC 8th) includes the extents or existence of tumor diameter, vascular invasion, solitary or multiple tumors, perforation of the visceral peritoneum, and direct invasion of local extrahepatic structures. Nodal and metastasis categories’ conditions between the two staging systems were similar, so we counted them together. Only one patient was diagnosed with T1b, that is, had a tumor size larger than 5 cm and without vascular invasion, T1a and T1b tumors were combined in the following study.

**Table 1:** Clinicopathologic characteristics of study patients.

Variable types	Variable name		Entire dataset (n=1,390)
**Basic information and medical record**	Age, year, median (IQR)		55 (46–62)
	Sex, female, n (%)		939 (67.6%)
	Blood type, n (%)	A	413 (29.7%)
		B	379 (27.3%)
		AB	136 (9.8%)
		O	462 (33.2%)
	Jaundice, n (%)		160 (11.5%)
	History of stone, n (%)		264 (19.0%)
	History of tumor, n (%)		101 (7.3%)
	HBV, n (%)		316 (22.7%)
	Smoking, n (%)		374 (26.9%)
**Tumor information**	Tumor size, cm, median (IQR)		6.0 (4.0–8.2)
	T classification (AJCC 8th), n (%)	T1a and T1b	277 (19.9%)
		T2	186 (13.4%)
		T3	544 (39.1%)
		T4	383 (27.6%)
	N classification (AJCC 8th), n (%)	N0	966 (69.5%)
		N1	424 (30.5%)
	M classification (AJCC 8th), n (%)	M0	1,214(87.3%)
		M1	176 (12.7%)
	TNM stage (AJCC 8th), n (%)	IA and IB	237 (17.1%)
		II	147 (10.5%)
		IIIA	376 (27.1%)
		IIIB	456 (32.8%)
		IV	174 (12.5%)
	T classification (LCSGJ), n (%)	T1	28 (2.0%)
		T2	562 (40.4%)
		T3	540 (38.9%)
		T4	260 (18.7%)
	TNM stage (LCSGJ), n (%)	I	27 (1.9%)
		II	472 (34.0%)
		III	317 (22.8%)
		IVA	108 (7.8%)
		IVB	466 (33.5%)
	Excision, n (%)	R0	1,253 (90.1%)
		R1	54 (3.9%)
		R2	83 (6.0%)
**Laboratory results**	CA19-9, U/ml, median (IQR)		55.0 (18.0–490.4)
	γ-GT, U/l, median (IQR)		84.0 (44.0–177.0)
	Alb, g/l, median (IQR)		42.1 (39.3–44.6)
	ALT, U/l, median (IQR)		27.1 (17.7–44.8)
	ALP, U/l, median (IQR)		110.0 (83.0–153.0)
	PA, mg/l, median (IQR)		212.0 (170.0–257.0)
	AST, U/l, median (IQR)		29.0 (21.9–42.3)
	CEA, μg/l, median (IQR)		2.9 (1.6–6.0)
	AFP, μg/l, median (IQR)		3.5 (2.29.0)
	DBIL, umol/l, median (IQR)		4.7 (3.5–6.5)
	TBIL, umol/l, median (IQR)		12.6 (9.5–17.2)

IQR, interquartile range; HBV, hepatitis B virus; AJCC 8th, the 8th edition of the American Joint Committee on Cancer staging system; LCSGJ, the Liver Cancer Study Group of Japan staging system; CA19-9, carbohydrate antigen; γ-GT, γ-glutamyltranspeptidase; Alb, albumin; ALT, alanine aminotransferase; ALP, alkaline phosphatase; PA, prealbumin; AST, aspartate aminotransferase; CEA, carcinoembryonic antigen; AFP, alpha-fetoprotein; DBIL, direct bilirubin; TBIL, total bilirubin.

### Selection and Comparison of Prognostic Factors

The IS of variables, most relevant to patient OS for 3 years were calculated by XGBoost, RF, and GBDT, the top 20 important variables selected from which were assembled in **Table 2**. Then we extracted the intersection of the above variables, and the retained 15 important variables were ALP, γ-GT, N, T, Alb, tumor size, AST, DBIL, TBIL, PA, ALT, AFP, CEA, CA19-9, and age. Among the variables, IS of T staging of AJCC 8th were higher than that of LCSGJ staging system, therefore T (AJCC 8th) was adopted and used “T” as a general name in the following analysis. Variables screened by machine learning participated in developing the Cox proportional hazard regression model. **Table 3** counted the variables in training cohort (n=1,112) used for modeling and the internal validation cohort (n=278) used for verification. The median survival time (months) of training cohort and internal validation cohort was 15.6 (IQR: 7.9–27.7) and 15.3 (IQR: 7.1–27.4), respectively. The data distribution among all factors in cohorts had relative equilibrium (*p*>0.05).

**Table 2:** The important variables calculated by XGBoost, random forest (RF), and gradient boosted decision tree (GBDT), and their intersection variables.

No.	XGBoost	IS	RF	IS	GBDT	IS	Intersection variables
1	ALP	0.0792	CA19-9	0.0948	CA19-9	0.1201	ALP
2	Alb	0.0774	ALP	0.0744	T (AJCC8th)^a^	0.1023	γ-GT
3	Age	0.0738	PA	0.0711	ALP	0.0897	N
4	CA19-9	0.0725	γ-GT	0.0645	PA	0.0788	T
5	CEA	0.0724	Tumor size	0.0643	ALT	0.0693	Alb
6	AFP	0.0719	CEA	0.0624	γ-GT	0.0646	Tumor size
7	ALT	0.0707	AST	0.0615	Tumor size	0.0600	AST
8	PA	0.0671	AFP	0.0591	AFP	0.0593	DBIL
9	TBIL	0.0660	Alb	0.0591	CEA	0.0535	TBIL
10	γ-GT	0.0659	ALT	0.0582	Alb	0.0506	PA
11	AST	0.0653	T (AJCC8th)^a^	0.0549	Age	0.0456	ALT
12	Tumor size	0.0617	TBIL	0.0531	DBIL	0.0435	AFP
13	DBIL	0.0615	Age	0.0530	AST	0.0394	CEA
14	T (AJCC8th)^a^	0.0243	DBIL	0.0521	TBIL	0.0388	CA19-9
15	T (LCSGJ)^a^	0.0122	T (LCSGJ)^a^	0.0264	N	0.0329	Age
16	N	0.0114	N	0.0176	T (LCSGJ)^a^	0.0194	
17	M	0.0084	Smoking	0.0098	History of stone	0.0057	
18	Smoking	0.0069	Blood type B	0.0094	M	0.0055	
19	Blood type A	0.0065	Gender	0.0085	History of tumor	0.0036	
20	History of stone	0.0058	Blood type A	0.0082	Blood type AB	0.0036	

^a^Since the importance score of T (AJCC 8th) in the three models is greater than T (LCSGJ), stage T is merged and only expressed as T in the intersection variables and following article.

XGBoost, eXtreme Gradient Boosting; RF, random forest; GBDT, gradient boosted decision tree; IS, importance score; ALP, alkaline phosphatase; Alb, albumin; CA19-9, carbohydrate antigen 19-9; CEA, carcinoembryonic antigen; AFP, alpha-fetoprotein; ALT, alanine aminotransferase; PA, prealbumin; TBIL, total bilirubin; γ-GT, γ-glutamyltranspeptidase; AST, aspartate aminotransferase; DBIL, direct bilirubin.

**Table 3:** Variable characteristic statistics of the training cohort and internal validation cohort.

Variables	Training cohort (n=1,112)	Internal validation cohort (n=278)	*p value*
Age, years, median (IQR)	55 (46–62)	55 (45–62)	0.412
T, n (%)			0.111
T1a and T1b	225 (20.2%)	52 (18.7%)	
T2	154 (13.9%)	32 (11.5%)	
T3	438 (39.4%)	106 (38.1%)	
T4	295 (26.5%)	88 (31.7%)	
N, n (%)			0.046^*^
N0	787 (70.8%)	179 (64.4%)	
N1	325 (29.2%)	99 (35.6%)	
Tumor size, cm, median (IQR)	5.8 (4.0–8.1)	6.0 (4.1–8.2)	0.209
CA19-9, U/ml, median (IQR)	54.7 (18.4–489.1)	58.3 (16.4–499.8)	0.380
γ-GT, U/l, median (IQR)	85.0 (44.8–178.4)	79.0 (43.0–174.5)	0.253
Alb, g/l, median (IQR)	42.1 (39.4–44.7)	41.8 (38.6–44.3)	0.046^*^
ALT, U/l, median (IQR)	26.9 (17.6–44.8)	28.4 (18.2–44.7)	0.287
ALP, U/l, median (IQR)	109.0 (83.0–156.0)	113.0 (82.0–148.0)	0.250
PA, mg/l, median (IQR)	212.0 (170.0–259.0)	210.0 (162.3–250.0)	0.070
AST, U/l, median (IQR)	28.8 (21.9–41.1)	29.5 (22.2–45.7)	0.090
CEA, μg/l, median (IQR)	3.0 (1.7–5.9)	2.7 (1.6–6.7)	0.323
AFP, μg/l, median (IQR)	3.6 (2.2–9.2)	3.3 (2.3–8.6)	0.338
DBIL, μmol/l, median (IQR)	4.7 (3.5–6.4)	4.7 (3.5–6.7)	0.173
TBIL, μmol/l, median (IQR)	12.6 (9.4–17.1)	12.7 (9.7–17.5)	0.299

T and N indicates the staging results of AJCC 8th; IQR, interquartile range; CA19-9, carbohydrate antigen 19-9; γ-GT, γ-glutamyltranspeptidase; Alb, albumin; ALT, alanine aminotransferase; ALP, alkaline phosphatase; PA, prealbumin; AST, aspartate aminotransferase; CEA, carcinoembryonic antigen; AFP, alpha-fetoprotein; DBIL, direct bilirubin; TBIL, total bilirubin. ^*^p < 0.05.

The data sets in **Table 3** were used to perform the Cox regression model, and further screened through backward stepwise regression (*p*<0.05). The results of backward stepwise regression are demonstrated in **Table 4**. The natural logarithmic transformation was applied on the continuous variables to avoid deviation of data distribution. Multivariate analysis by stepwise regression revealed that T classification of AJCC 8th (HR, 1.204; 95% CI, 1.142–1.270), N (HR, 1.927; 95% CI, 1.655–2.243), ln (CEA) (HR, 1.158; 95% CI, 1.098–1.221), ln (CA19-9) (HR, 1.127; 95% CI, 1.085–1.171), ln (AFP) (HR, 1.057; 95% CI, 1.019–1.096), and ln (PA) (HR, 0.830; 95% CI, 0.714–0.964) were determined to be independent predictors of 3-year OS in ICC patients.

**Table 4:** Multivariate regression analysis in the training cohort (n=1,112).

Variables	β	SE (β)	Waldχ^2^	HR	95% CI	*p-value*
T	0.186	0.027	6.844	1.204	1.142–1.270	<0.001^***^
N	0.656	0.078	8.433	1.927	1.655–2.243	<0.001^***^
ln (CEA)	0.147	0.027	5.426	1.158	1.098–1.221	<0.001^***^
ln (CA19-9)	0.120	0.019	6.166	1.127	1.085–1.171	<0.001^***^
ln (AFP)	0.055	0.019	2.972	1.057	1.019–1.096	0.003^**^
ln (PA)	−0.187	0.077	−2.439	0.830	0.714–0.964	0.015^*^

T and N indicates the staging results of AJCC 8th; β, regression coefficient; SE, standard error; HR, hazard ratio; CI, confidence interval; CEA, carcinoembryonic antigen; CA19-9, carbohydrate antigen 19-9; AFP, alpha-fetoprotein; PA, prealbumin. 0.01 < ^*^p < 0.05, 0.001 < ^**^p < 0.01, ^***^p < 0.001.

### Variable Selection Methods Comparison

**Figure 2:**
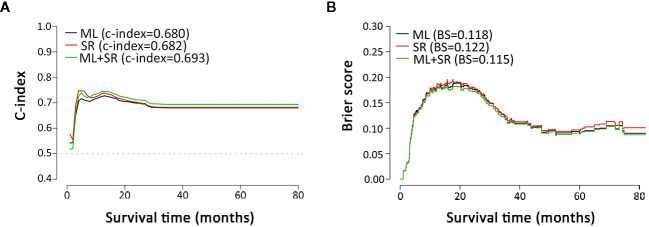
Metrics comparison of models based on different multivariate analysis approaches. **(A, B)** are C-index and brier score comparisons of models based on multivariate analysis by ML, SR, and ML+SR, respectively. ML, machine learning; SR, stepwise regression.

The Cox regression models with stepwise selection were commonly used in similar studies to select variables, which significantly associated with the prognostic outcome after ICC resection. To verify whether the variable selection incorporated machine learning algorithms can improve the model accuracy or not, we performed three approaches for comparison: only by Cox proportional hazards model with backward stepwise regression (namely SR), only by machine learning (namely ML), and combining both methods (SR+ML) (**Figure 2**). By establishing the survival prediction models, the C-index (**Figure 2A**) and BS (**Figure 2B**) of the above three approaches were obtained, and the results demonstrated that SR+ML (C-index, 0.693; BS, 0.115) had better performance in the most of survival time than only ML and only SR. Therefore, machine learning was proven to capture the prognostic predictors of postoperative outcome more accurately during variable processing, consequently improving the prediction performance of the model. The influenced factors selected *via* only SR including: sex, age, history of stone, smoking habit, HBV, T, N, M, CA19-9, PA, CEA, DBIL, TBIL, excision, and the blood type A. The variables screening results of SR *via* Cox analysis were summarized in **Supplemental Table 2**.

### Establishment and Evaluation of Eastern Hepatobiliary Surgery Hospital-Intrahepatic Cholangiocarcinoma Scoring System

Based on the Cox regression, the range of the prognostic index for each individual is from −1.2 to 2.4. In order to adjust the score in our proposed scoring system into positive, we obtained the EHBH-ICC scoring formula as follows:

$$EHBH–ICC\_score=10\times \left(\begin{array}{l} 1.2+0.186\times T+0.656\times N+0.147\times 1n\left(CEA\right)+0.120\times 1n\left(CA19–9\right)+\\ 0.055\times 1n\left(AFP\right)–0.187\times 1n\left(PA\right) \end{array}\right)$$

**Figure 3:**
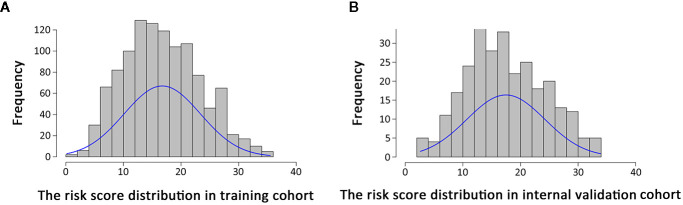
Distribution of risk scores in patients using Eastern Hepatobiliary Surgery Hospital-intrahepatic cholangiocarcinoma (EHBH-ICC) scoring system. **(A, B)** are risk score distributions in training cohort (n=1,112, median=16.3) and internal validation cohort (n=278, median=17.0), respectively.

**Figure 4:**
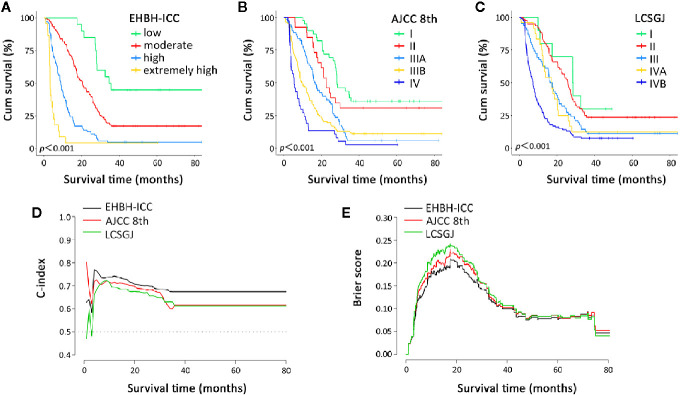
Overall survival curves and prognostic performance indicator curves in the Eastern Hepatobiliary Surgery Hospital-intrahepatic cholangiocarcinoma (EHBH-ICC), American Joint Committee on Cancer (AJCC) 8th, and the Liver Cancer Study Group of Japan (LCSGJ) staging systems. **(A–C)** depict the overall survival according to the three staging systems in internal validation cohort, all log rank *p*<0.001. **(D, E)** present the C-index and brier score change in long-term survival, respectively.

Histograms of survival risk score distribution for training cohort and internal validation cohort were built based on our EHBH-ICC score (**Figures 3A, B**). According to the score distribution, we divided patients into four risk groups: low (0–10), moderate (11–20), high (21–30), and extremely high (>30). The median risk scores in training and internal validation cohorts were 16.3 and 17.0, respectively. **Figure 4A** displays the good prognostic stratification for patients between stages in internal validation cohort (log rank *p*<0.001).

### Comparison of Predictive Accuracy for Overall Survival in Eastern Hepatobiliary Surgery Hospital-Intrahepatic Cholangiocarcinoma, American Joint Committee on Cancer 8th and the Liver Cancer Study Group of Japan Staging System

Further, we made a comparison of the EHBH-ICC staging system with AJCC 8th and the LCSGJ staging systems. Since time-to-mortality and time-to-event were crucial to interpret the results, **Figures 4A–C** depict the Kaplan-Meier curves of the three different staging systems. All of three systems in our study appeared a progressive decrease in OS during the study period. The log-rank test proved that all these staging methods have *p*<0.001.

The discrimination ability and prediction performance of EHBH-ICC score model in internal validation cohort and external validation cohort were respectively indicated with higher C-index of 0.693 (95% CI, 0.663–0.723) and 0.671 (95% CI, 0.602–0.740) than the AJCC 8th and LCSGJ staging systems, which were then confirmed with lower probability calibration of BS (0.103 in internal validation cohort and 0.169 external validation cohort). Detailed C-index and BS results are presented in **Table 5** and **Figures 4D, E**. The model evaluation results show that the EHBH-ICC score was the most precise in predicting the survival after resection in this study.

**Table 5:** The comparison of Eastern Hepatobiliary Surgery Hospital (EHBH)-intrahepatic cholangiocarcinoma (ICC), American Joint Committee on Cancer (AJCC) 8th and the Liver Cancer Study Group of Japan (LCSGJ) staging system in internal and external validation cohorts.

Cohorts	Models	C-index (95% CI)	BS
**Internal validation**	**EHBH-ICC**	**0.693 (0.663–0.723)**	**0.103**
	AJCC 8th	0.675 (0.642–0.708)	0.110
	LCSGJ	0.665 (0.632–0.698)	0.114
**External validation**	**EHBH-ICC**	**0.671 (0.602–0.740)**	**0.169**
	AJCC 8th	0.648 (0.578–0.718)	0.198
	LCSGJ	0.539 (0.455–0.623)	0.189

CI, confidence interval; BS, brier score; EHBH-ICC, the prognostic scoring system for postoperative intrahepatic cholangiocarcinoma proposed by Eastern Hepatobiliary Surgery Hospital; AJCC 8th, the 8th edition of the American Joint Committee on Cancer staging system; LCSGJ, the Liver Cancer Study Group of Japan staging system. The discrimination ability and prediction performance of EHBH-ICC score model in internal validation cohort and external validation cohort were respectively indicated with higher C-index of 0.693 (95% CI, 0.663–0.723) and 0.671 (95% CI, 0.602–0.740) than the AJCC 8th and LCSGJ staging systems, which were then confirmed with lower probability calibration of BS (0.103 in internal validation cohort and 0.169 external validation cohort).

## Discussion

ICC is the second most common primary hepatic malignancies after HCC with increasing incidence and mortality worldwide (21, 22). Hepatectomy is considered as the mainstay of curative option for ICC (23). Accurate tumor staging provides the prognostic details, evaluates the risk level appropriately, as well as assists the choice of adjuvant therapeutic options.

At present, the most commonly used staging systems for ICC are the TNM classification systems, among which, the AJCC 8th and LCSGJ are widely approbatory. With relentless efforts of AJCC to improve the prognostic staging of ICC, there are still research evidences that it is inadequate. T1b with single lesion larger than 5 cm without vascular invasion in AJCC was often rare in clinical treatments. And some recent studies indicated that stage II and stage IIIA for ICC patients in AJCC edition failed to show significant prognostic differentiation. Survival time for intrahepatic metastases was sometimes lower than in patients with serous membrane protruding tumors; however, these patients were only at T2 stage. Some recent studies assessed the prognostic performance of the 7th and 8th edition versions of AJCC staging system, proving that there was no remarkable improvement in overall prognostic discrimination, especially in the staging of T3 category (14, 24, 25). While the LCSGJ focuses on the HCC which has distinct differences in biological behaviors and postoperative outcomes. Some modified staging systems for resectable ICC reserved the prognostic factors in TNM classification or combined these two systems as one of the predictors (19, 26). In our investigation, we analyzed the diagnoses of both staging systems above as separate independent variables. We hypothesized that pathology factors are important prognostic factors for postoperative ICC patients but are only partially relevant. Our study was based on multi-dimensional clinical real-world data in relatively larger population, thus we could seek factors affecting postoperative survival of ICC patients with a wider perspective.

We derived 15 important factors by three algorithms concurrently (**Table 2**), and further identified T (AJCC 8th) and N classifications, CEA, CA19-9, AFP, PA as the prognostic predictive factors. Multiple potential tumor biomarkers have been used in evaluating the prognosis of ICC (27–29). For now, many researches have constructed some new assessment systems with diagnostic biomarkers to predict the survival of patients, such as CA19-9, AFP, CEA, ALP, and PA (17, 19, 30). These factors were confirmed by our results and were involved in the outcome scoring of ICC patients. Serum CA 19-9 and CEA were most investigated in prognosis of ICC (17, 18, 31). Jaklitsch et al. had proven that the inclusion of preoperative CA 19-9 and CEA in AJCC and LCSGJ staging systems improved the prognostic survival prediction after resection for ICC (32). Serum AFP is a widely used tumor marker of HCC (33), and the positive serum AFP (>20 ng/ml) is seen in approximately 19% of ICC patients (34). Zhou et al. showed that the lymph node metastasis rate was low in ICC patients with positive AFP (35). PA generated by liver is commonly regarded as a sensitive marker of nutritional status. A study reported that patients with lower PA have poorer outcomes in ICC (19), which is consistent with our result that PA level is negatively associated with the score. Compared with pathological factors, clinical parameters are easier to obtain and can also provide valuable reference. In our EHBH-ICC scoring system, the diagnosis of T and N and the laboratory results can be directly substituted into the calculation to obtain the corresponding risk level scores.

To our knowledge, our report is the first ICC staging method developed based on machine learning models. In recent years, machine learning-based methods are widely used in diagnosis, treatment and outcome prediction such as prostate cancer (36), renal cancer (37), non-small cell lung cancer (38), and cardiovascular event prediction (39). Machine learning can deal with different data types even if data are incomplete or incoherent comparing with traditional statistics. Many studies have demonstrated the advantages of machine learning algorithms over traditional statistical methods (40).

According to the EHBH-ICC scoring system, patients are divided into four survival risk grades (low to extremely high). This is a scoring approach to predict the outcome of resectable ICC in Chinese population. The other scoring approach, for instance, the Fudan scoring system was only conducted for 344 patients with multivariate Cox regression. Compared with the Fudan scoring system, the EHBH-ICC has different calculation methods and key prognostic factors. A similarity between Fudan scoring system and our system was the discovery and application of the prognostic value of readily available clinical parameters. Our ultimate validation methods of discrimination ability and performance were C-index and BS. The EHBH-ICC scoring system (C-index, 0.693; BS, 0.103) has more accurate prognostic prediction for ICC patients *via* comparison with the AJCC 8th and LCSGJ edition (**Figures 4D, E**).

In our study, patients’ tumor diversity was well reflected. With the continuously increasing sample size, the evaluation system will be more optimized to predict the prognosis of patients more accurately to make decision of the treatment. We cannot only obtain the proportion of risk factors in the prognosis of patients, but also accurately predict the prognosis of patients with the increasing score *via* machine learning.

However, there are limitations in our study. Our study is a retrospective study in one single center. More medical centers and samples could be added to optimize our evaluation system and solve the limitation. In conclusion, the EHBH-ICC scoring system shows good predictive ability for ICC patients who underwent surgical operation *via* evaluation and comparison with existing staging systems (the AJCC 8th and LCSGJ). The machine learning-based EHBH-ICC scoring system can effectively evaluate the ICC prognosis after resections and be used in clinical practice.

## Data Availability Statement

The original contributions presented in the study are included in the article/**Supplementary Material**; further inquiries can be directed to the corresponding authors.

## Ethics Statement

The protocol of this study has been approved by the Ethics Committee of the Eastern Hepatobiliary Surgery Hospital, and the informed consent has been exempted in the Ethical approval documents.

## Author Contributions

ZL and LY conceptualized the study. JS and ZW contributed to the methodology. JS and CZ conducted the formal analysis and investigation. ZL, YW, and XH wrote and prepared the original draft. FG and XJ provided the resources and supervised the study. All authors contributed to the article and approved the submitted version.

## Conflict of Interest

JS and FG were employed by company Beijing Medicinovo Technology Co., Ltd. YW and XH were employed by company Dalian Medicinovo Technology Co., Ltd.

The remaining authors declare that the research was conducted in the absence of any commercial or financial relationships that could be construed as a potential conflict of interest.
