# Supplementary material for: A Novel Prognostic Scoring System of Intrahepatic Cholangiocarcinoma With Machine Learning Basing on Real-World Data
Source: Front Oncol. 2021 Jan 20;10:576901. doi: 10.3389/fonc.2020.576901 (PMC7855854; doi:10.3389/fonc.2020.576901)
Supplement: Supplementary file 1 [file Table_1.docx]

# SUPPLEMENTARY MATERIAL

## Supplemental Table 1

Variable characteristic statistics of the training cohort and external validation cohort.

| **Variables** | **Training cohort (n=1112)** | **External validation cohort (n=42)** | ***p value*** |
| --- | --- | --- | --- |
| T, n (%) |  |  | 0.003^**^ |
| T1a & T1b | 225 (20.2%) | 2 (4.8%) |  |
| T2 | 154 (13.9%) | 13 (30.9%) |  |
| T3 | 438 (39.4%) | 18 (42.9%) |  |
| T4 | 295 (26.5%) | 9 (21.4%) |  |
| N, n (%) |  |  | <0.001^***^ |
| N0 | 787(70.8%) | 14 (33.3%) |  |
| N1 | 325(29.2%) | 28 (66.7%) |  |
| CA19-9, U/ml, median (IQR) | 54.7 (18.4-489.1) | 175.5 (21.3-1000) | 0.107 |
| PA, mg/l, median (IQR) | 212.0 (170.0-259.0) | 216.5 (120.8-263.0) | 0.183 |
| CEA, μg/l, median (IQR) | 3.0 (1.7-5.9) | 3.3 (1.7-11.4) | 0.300 |
| AFP, μg/l, median (IQR) | 3.6 (2.2-9.2) | 3.1 (2.1-4.7) | 0.094 |

*T, N indicates the stages of AJCC 8th; CA19-9, carbohydrate antigen 19-9; PA, prealbumin; CEA, carcinoembryonic antigen; AFP, alpha-fetoprotein. 0.001<^**^p<0.01, ^***^p<0.001.*

## Supplemental Table 2

Multivariate analysis of prognostic factors on overall survival performed by Cox proportional hazards models with stepwise regression.

| **Variables** | **HR** | **95% CI** | ***p value*** |
| --- | --- | --- | --- |
| Age | 0.824 | 0.702-0.968 | 0.018*^*^* |
| Sex | 0.993 | 0.987-0.999 | 0.030*^*^* |
| Smoking | 1.464 | 1.232-1.739 | ＜0.001*^***^* |
| History of stone | 0.781 | 0.660-0.924 | 0.004*^**^* |
| HBV | 1.214 | 1.017-1.450 | 0.032*^*^* |
| T | 1.211 | 1.147-1.280 | ＜0.001*^***^* |
| M | 1.633 | 1.381-1.932 | ＜0.001*^***^* |
| N | 1.523 | 1.240-1.870 | ＜0.001*^***^* |
| CA19-9 | 1.001 | 1.000-1.001 | ＜0.001*^***^* |
| PA | 0.999 | 0.998-1.000 | 0.019*^*^* |
| CEA | 1.001 | 1.001-1.002 | ＜0.001*^***^* |
| DBIL | 0.978 | 0.960-0.996 | 0.019*^*^* |
| TBIL | 1.017 | 1.003-1.032 | 0.021*^*^* |
| Resection type | 1.430 | 1.246-1.642 | ＜0.001*^***^* |
| Blood Type A | 1.184 | 1.025-1.368 | 0.022*^*^* |

*T, M, N indicates the stages of AJCC 8th; HR, hazard ratio; CI, confidence interval; HBV, hepatitis B virus; CA19-9, carbohydrate antigen 19-9; PA, prealbumin; CEA, carcinoembryonic antigen; DBIL, direct bilirubin; TBIL, total bilirubin. 0.01<^*^p<0.05, 0.001<^**^p<0.01, ^***^p<0.001.*
